# PRR11 is a prognostic biomarker and correlates with immune infiltrates in bladder urothelial carcinoma

**DOI:** 10.1038/s41598-023-29316-2

**Published:** 2023-02-04

**Authors:** Wenpeng Ni, Lijuan Yi, Xiaoru Dong, Mengjie Cao, Jinjuan Zheng, Qingling Wei, Chunlei Yuan

**Affiliations:** 1 Clinical Laboratory, Zhongshan Boai Hospital Affiliated to Southern Medical University, Zhongshan, 528400 Guangdong China; 2 Department of Comprehensive Ophthalmology, Zhongshan Aire Eye Hospital, Zhongshan, Guangdong China; 3 Department of Neurology, The Second Hospital of Hebei Medical University, Shijiazhuang, Hebei China; 4 The Second School of Clinical Medicine, Southern Medical University, Guangzhou, Guangdong China

**Keywords:** Cancer genomics, Tumour biomarkers, Tumour immunology, Urological cancer

## Abstract

Abnormal proline-rich protein 11 (PRR11) expression is associated with various tumors. However, there are few reports concerning PRR11 with prognostic risk, immune infiltration, or immunotherapy of bladder urothelial carcinoma (BLCA). This study is based on online databases, such as Oncomine, GEPIA, HPA, LinkedOmics, TIMER, ESTIMATE and TISIDB, and BLCA data downloaded from The Cancer Genome Atlas (TCGA) and Gene Expression Omnibus, we employed an array of bioinformatics methods to explore the potential oncogenic roles of PRR11, including analyzing the relationship between PRR11 and prognosis, tumor mutational burden (TMB), microsatellite instability, and immune cell infiltration in BLCA. The results depict that PRR11 is highly expressed in BLCA, and BLCA patients with higher PRR11 expression have worse outcomes. In addition, there was a significant correlation between PRR11 expression and TMB and tumor immune infiltration. These findings suggest that PRR11 can be used as a potential marker for BLCA patient assessment and risk stratification to improve clinical prognosis, and its potential regulatory mechanism in the BLCA tumor microenvironment and targeted therapy is worthy of further investigation.

## Introduction

Bladder cancer mainly includes urothelial (transitional cell carcinoma), squamous cell carcinoma, and adenocarcinoma. Among them, urothelial carcinoma is the most common, accounting for more than 90% of the total prevalence of bladder cancer^1^. It has been identified that cigarette smoke^2^, intake of aristolochic acid^3^, arsenic exposure^4,5^, and occupational exposure to aromatic amines^6,7^ have significant effects on tumorigenesis of BLCA. BLCA is the top ten cancer type and the second most prevalent genitourinary system tumor worldwide, with approximately 429,000 new cases and 165,000 deaths annually^8^. Despite advances in surgical techniques and postoperative management, the clinical outcomes of BLCA patients have not improved much over the past few decades due to the lack of sensitive markers^9^. Before diagnosing and treating BLCA, it is necessary to determine whether the cancer is muscle-invasive or non-muscle invasive. According to the clinical characteristics of the two, muscle-invasive cancer requires radical cystectomy, while non-muscle invasive cancer can be excised locally using minimally invasive surgery, followed by routine intravesical chemotherapy. The prognosis of 70% of cases with non-muscular invasive BLCA improves after treatment with transurethral resection (TURB) and intravesical chemotherapy, immunotherapy with Bacillus Calmette-Guérin^10^. However, 40% of treated patients will develop muscle-invasive BLCA within five years^11^. Patients with muscular invasive BLCA have a higher risk of long-distance metastasis and a worse prognosis than those with non-muscular invasive BLCA^12^. Thus, discovering specific early detection markers and therapeutic targets is the key to improving the survival rate of BLCA patients.

Immune cell infiltration is an important component of the tumor microenvironment (TME), and it plays a significant role in the initiation, progression, and clinical treatment of tumors^13–15^. Changes in the immune microenvironment may affect clinical outcomes in various tumors, such as melanoma, lung cancer, breast cancer, and muscle-invasive bladder cancer^16^. It has been reported that immune cells and related genes in the BLCA microenvironment promote tumor progression^19^, and the infiltrating characteristics of macrophages in BLCA may affect T-cell tolerance, thus altering patient outcomes^17,18^. Considering the current development of various cellular immunotherapy and targeted therapies as well as their clinical application trends, it is instructive to clarify the characteristics of immune infiltration and internal regulation mechanisms of various types of neoplastic disease.

The PRR11 gene on chromosome 17q22 encodes a proline-rich protein with a zinc finger domain and a bivalent nuclear localization signal^20^. Since its discovery in 2013, the relationship between this gene and tumors has been getting closer, and reports have revealed that it is related to the poor outcomes of various tumor types^21^, such as ovarian carcinoma^22^, breast cancer^23^, non-small cell lung cancer^24^, colorectal cancer^25^, and pancreatic cancer^26^. In addition, data indicate that it is involved in the epithelial-mesenchymal transition (EMT) process^27^. Therefore, we hypothesize that PRR11 may have diagnostic, prognostic, and targeted therapy applications in various tumor types. When performing an in-depth analysis of the full-gene difference of BLCA, our team discovered that PRR11 is a gene with a notable difference. Lin et al*.* also suggested that PRR11 may increase risk scores in bladder cancer patients^28^. Because there are few reports on the relationship between PRR11 and BLCA, we have conducted further research on this in the follow-up.

In this study, we analyzed and verified the expression of PRR11 in BLCA using GEPIA, Oncomine, TCGA, and other databases before calculating its significance in the clinical prognosis of patients using various statistical methods. Using Timer, ESTIMATE, TISIDB, and LinkedOmics database, we also clarified the primary biological function of PRR11 and its potential impact on BLCA immune infiltration. In conclusion, our findings suggested a significant prognostic value of PRR11 and a potentially promising target for immunotherapeutic strategies in BLCA.

## Materials and methods

### Data acquisition and processing

We used the TCGA database (https://genome-cancer.ucsc.edu/) to collect RNA-seq data and clinical information from 411 BLCA projects, including 19 cases of matching adjacent tissues. The downloaded data format is level 3 HTSeq-fragments per kilobase per million (FPKM). We also downloaded TPM (transcripts per million) format RNA-seq data of 9 normal human bladder tissues in Genotype-Tissue Expression (GTEx) database that uniformly processed by Toil process from UCSC Xena (https://xenabrowser.net/datapages/)^29^. The gene expression profiling data sets, GSE48276(n = 116) and GSE13507(n = 256), were obtained from GEO database (https://www.ncbi.nlm.nih.gov/gds).

We used R (v4.1.0) to generate the receiver operating characteristic (ROC) curve and analyze the expression of PRR11 in 19 paired samples in TCGA-BLCA dataset, and the pROC and “ggplot2” R package were used for visualization. The RNAseq data in the FPKM format of the paired samples were compared after the log2 conversion. The “survival” package was used for statistical analysis of survival data, the “survminer” package for visualization. The expression of PRR11 protein in bladder tissue was analyzed based on the immunohistochemistry data from HPA (Human Protein Atlas) database (https://www.proteinatlas.org/).

### Genome-wide analysis of differential gene

Limma package (version: 3.40.2) of R software was used to study the differential expression of mRNAs in BLCA . The adjusted P-value was analyzed to correct for false positive results in TCGA or GTEx. “Adjusted P < 0.05 and Log (Fold Change) > 1 or Log (Fold Change) <  − 1” were defined as the thresholds for the screening of differential expression of mRNAs (Supplementary Fig. 1). We selected genes with Log (Fold Change) > 2.5, P and adjusted P < 0.001 from the screening results, then set PRR11 as the research target.

### Oncomine database analysis

We used Oncomine (https://www.oncomine.org/resource/login.html) to identify the expression level of PRR11 in various tumors^30^. The threshold was set at a P value of 1e-5, a multiple change of 1.5 and the gene rank of top 5%.

### GEPIA database analysis

Gene expression profiling interactive analysis (GEPIA) is an interactive web to analyze the RNA sequencing expression, including 9736 tumors and 8587 normal samples from TCGA and the GTEx projects (http://gepia.cancer-pku.cn/index.html)^31^. Here, we used GEPIA to re-analyze and verify the expression of PRR11, and produced a total survival (OS) map of PRR11 related genes based on log-rank test in BLCA. In addition, we explored the correlation between PRR11 expression and gene markers of tumor infiltrating immune cells through GEPIA database^32^, and produced the scatter plots of PRR11 expression between a pair of user defined genes in BLCA.

### Correlation of PRR11 expression with tumor mutation burden, tumor microsatellite instability, and mismatch repair gene expression

TMB and MSI scores were determined for all samples based on mRNA-seq data downloaded from TCGA (https://tcga.xenahubs.net), and Spearman’s correlation analysis was used to describe the correlation. Results are presented as scatter plot, produced by the R-package “ggstatspolt”. Expression profile data from TCGA were used to evaluate the levels of the mismatch repair (MMR) genes, including MutL homologous gene (MLH1), MutS homologous gene 2 (MSH2), MSH6, postmeiotic segregation increased 2 (PMS2), epithelial cell adhesionmolecule (EPCAM), in BLCA and determined the correlation between levels of MMR gene expression and PRR11. Data were visualized as a heatmap by the R-packages "ggstataplot".

### Relationship between PRR11 and immunity

The correlation between PRR11 expression and immune cell infiltration in BLCA was explored by a gene module in the Tumor Immune Estimation Resource (TIMER) database (https://cistrome.shinyapps.io/timer/)^33^. The TIMER database contains 32 cancers from the TCGA. The immune cells in the TIMER database include CD4^+^ T cells, CD8^+^ T cells, B cells, neutrophils, macrophages, and dendritic cells.

The TISIDB database (http://cis.hku.hk/TISIDB) is a web portal for tumour and immune system interaction, which integrates multiple heterogeneous data types. They pertain to 988 reported immune-relatedanti-tumour genes, high-throughput screening techniques, molecular profiling and para-cancerous multi-omics data, as well as numerous resources for immunological data gathered from seven public databases^34^. In this study, TISIDB provided us associations for PRR11 with Th1 and Th2 lymphocytes.

Estimation of STromal and Immune cells in MAlignant Tumor tissues using Expression data (ESTIMATE) database (https://bioinformatics.mdanderson.org/public-software/estimate/) is an algorithm using gene expression data to infer tumor purity and the degree of infiltration of immune cells into tumors^35^. The immunescore and tumorpurity score of each patient in datasets was calculated by the R package “estimate”, and visualized by "ggplot2" R package.

### Linked omics database analysis

LinkedOmics is publicly available portal that includes multi-omics data from all 32 TCGA Cancer types (http://www.linkedomics.org/login.php)^36^. The differentially expressed genes related to PRR11 were screened from the TCGA BLCA cohort through the LinkFinder module in the database, and the correlation of results was tested by the Spearman test and presented respectively in scatter plot and heat maps. Function module analysis of Kyoto Encyclopedia of Genes and Genomes (KEGG) pathways was performed by the gene set enrichment analysis (GSEA) in the LinkInterpreter module^37–39^.

### Statistical analysis

Wilcoxon rank sum test, Chi-square test, Fisher exact test and logistic regression were used to analyze the relationship between clinicopathological characteristics and PRR11 expression. The Kaplan–Meier method was used to calculate the OS and disease-free survival (DFS). Cox proportional hazards model was used for univariate and multivariate analysis to estimate the association between PRR11 expression and clinical characteristics, OS. P value less than 0.05 is considered statistically significant. SPSS15.0 was used for statistical analysis.

### Ethical approval

TCGA and GEO belong to public databases. The patients involved in the database have obtained ethical approval. Users can download relevant data for free for research and publish relevant articles. Our study is based on open source data, so there are no ethical issues and other conflicts of interest.

## Results

### Increased PRR11 expression in BLCA

**Figure 1:**
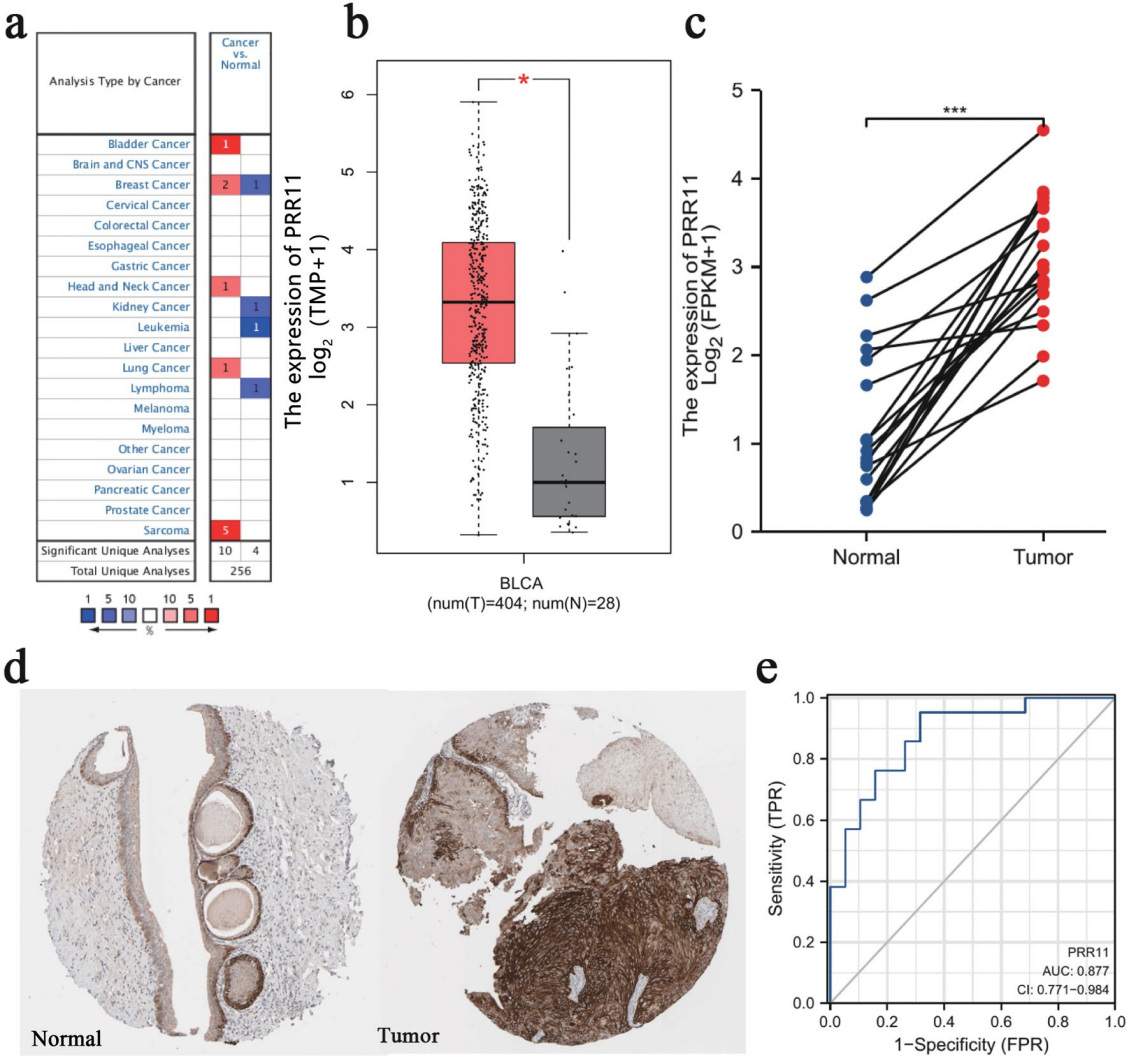
PRR11 expression is increased in BLCA. (**a**) Increased or decreased PRR11 in datasets of different cancers compared with normal tissues in the Oncomine database. Red represents high expression, blue represents low expression, and the shade of color depends on the degree of expression. (**b**) PRR11 expression in BLCA from TCGA database were determined by GEPIA. (**c**) The expression of PRR11 in 19 pairs of BLCA tissues and matched non-cancer tissues from the TCGA database. (**d**) The expression of PRR11 in normal and BLCA tissue sections from the HPA database. (**e**) ROC curve showed the efficiency of PRR11 expression level to distinguishing BLCA from non-tumor tissue in TCGA database. X-axis represents false positive rate, and Y-axis represents true positive rate. *P < 0.05, ***P < 0.001.

We initially used the Oncomine database to analyze the expression differences of PRR11 mRNA between cancer and normal tissues. The analysis demonstrated that the expression level of PRR11 mRNA was significantly increased in BLCA (Fig. 1a), which was also verified in the GEPIA database (Fig. 1b). PRR11 is also up-regulated in other types of cancer, such as breast, head and neck, and lung cancer, and its expression in sarcoma is particularly striking, which may be a new discovery. We then used 19 pairs of BLCA tissues and matched non-cancer tissues from the TCGA database to demonstrate that the comparison results were still accurate (Fig. 1c). Through the HPA database, we confirmed that the expression level of PRR11 protein in BLCA is still greater than that of normal tissues (Fig. 1d). In addition, we used the ROC curve to analyze the potential effectiveness of distinguishing BLCA from non-tumor tissues by PRR11 expression (Fig. 1e). The results demonstrated that the area under the curve (AUC) of PRR11 was 0.877, indicating that PRR11 can be used as an ideal biomarker to differentiating BLCA from non-tumorous tissues.

### Prognostic potential of PRR11 in BLCA

**Figure 2:**
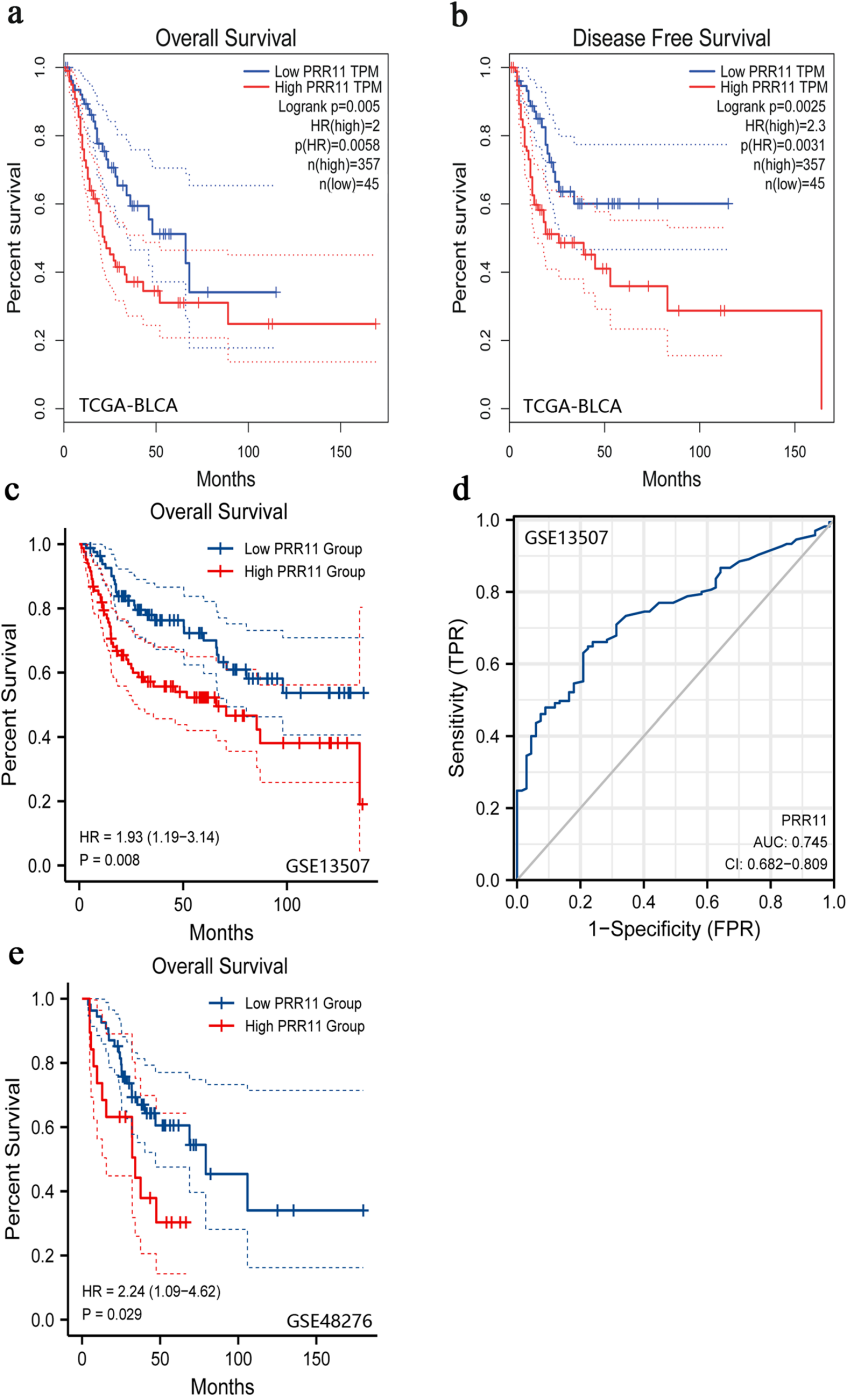
Kaplan‐Meier survival curves were used to analyze the correlation between gene alterations in PRR11 and OS, as well as the RFS of BLCA patients. In TCGA-BLCA dataset, high PRR11 expression was correlated with poor OS (**a**) and RFS (**b**). Patients with higher PRR11 expression level had higher OS time in the GSE13507 (**c**) and GSE48276 dataset (**e**). (**d**) ROC curve showed the efficiency of PRR11 expression level to distinguishing BLCA from non-tumor tissue in GSE48276 dataset. X-axis represents false positive rate, and Y-axis represents true positive rate.

We selected the optimal cut-off value of PRR11 (1.6025) based on the results of the ROC curve and divided 402 BLCA samples from the TCGA database into high expression and low expression groups. Kaplan–Meier analysis was used to explore the correlation between PRR11 expression and OS and DFS of BLCA patients. The results revealed that the poor prognosis in BLCA was significantly correlated with the high expression of PRR11 (Fig. 2a,b). In addition, the two datasets GSE48276 and GSE13507 were used to further validate the prognostic value of PRR11 (Fig. 2c–e). These results indicated that PRR11 has considerable prognostic value for BLCA.

### Correlation of PRR11 expression with clinicopathological factors in BLCA

The characteristics of samples were displayed in Table 1, in which 411 cases of BLCA with both clinical and gene expression data were collected from the TCGA database. The association between the level of PRR11 expression and the clinicopathological characteristics of BLCA patients was evaluated using the Chi-square test or Fisher's exact test, or Rank sum test. The results depicted that the expression level of PRR11 was significantly correlated with the patient's age, race, and pathological grade. In addition, the Logistic regression method demonstrated that PRR11 expression level is significantly correlated with pathological grade ( Table 2).

**Table 1:** Correlation between PRR11 expression and clinicopathological characteristics in BLCA.

Characters	Level/Classification	Low expression of PRR11	High expression of PRR11	P	TEST
n		46	365		
Age (years) n (%)	> 60	25 (54.3)	278 (76.2)	0.002	Chi-square
n	≦60	21 (45.7)	87 (23.8)		
Gender n (%)	Male	37 (80.4)	266 (72.9)	0.18	Chi-square
	Female	9 (19.6)	99 (27.1)		
Race n (%)	White	31 (67.4)	296 (81.1)	< 0.001	Fisher’s Exact
	Black or African	1 (2.2)	22 (6.0)		
	American				
	Asian	14 (30.4)	30 (8.2)		
T stage n (%)	T1	1 (2.2)	3 (0.8)	0.645	Rank sum
	T2	16 (34.8)	103 (28.2)		
	T3	15 (32.6)	180 (49.3)		
	T4	9 (19.6)	50 (13.7)		
N stage n (%)	N0	28 (60.9)	210 (57.5)	0.675	Rank sum
	N1	4 (8.7)	42 (11.5)		
	N2	8 (17.4)	68 (17.8)		
	N3	1 (2.2)	7 (1.9)		
M stage n (%)	M0	30 (65.2)	169 (46.3)	0.165	Rank sum
	M1	0 (0)	11 (3.0)		
Grade n (%)	Low	9 (19.6)	12 (3.3)	< 0.001	Rank sum
	High	36 (78.3)	351 (96.2)

**Table 2:** PRR11 expression associated with clinicopathologic characteristics (logistic regression).

Characteristics	HR	95%CI	P value
T stage(T1&T2 vs. T3&T4)	0.223	0.982–1.591	0.7
N stage(N0 vs. N1&N2&N3)	0.088	0.859–1.388	0.473
M stage(M0 vs. M1)	0.086	0.567–2.095	0.797
Grade(Low vs. High)	1.506	2.506–8.119	< 0.001

### Cox univariate and multivariate analysis of prognostic factors in BLCA

**Figure 3:**
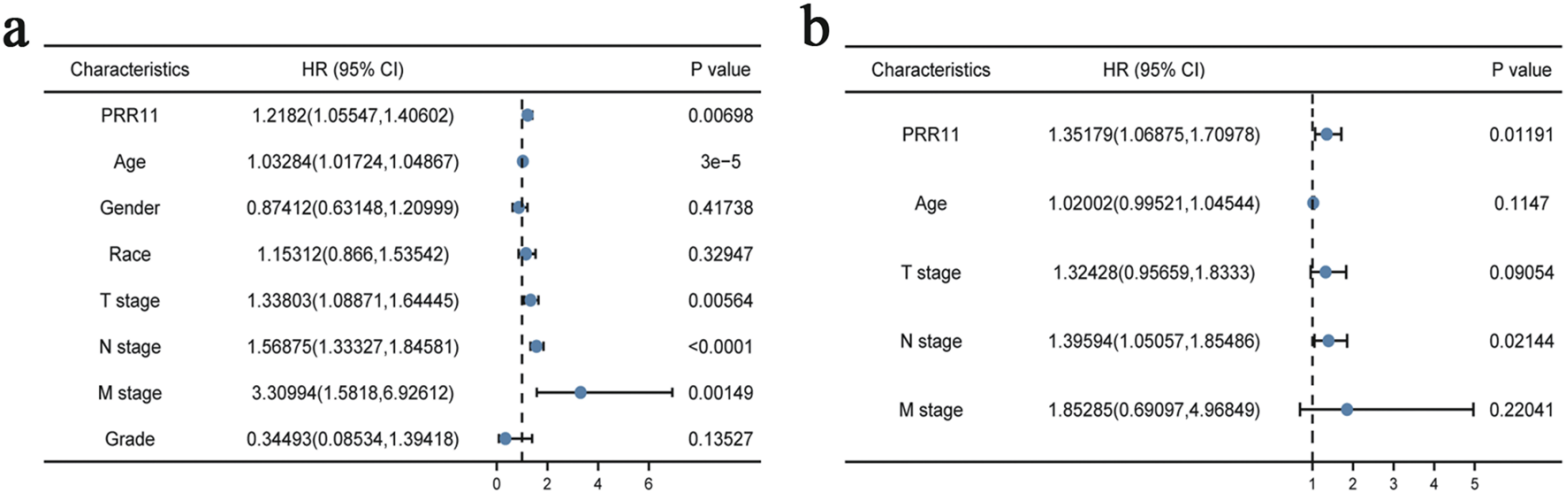
Univariate (**a**) and multivariate (**b**) regression analysis of PRR11 and other clinicopathologic parameters with OS in BLCA patients.

Figure 3a displays the results of Cox univariate and multivariate analysis of OS in BLCA. The variables with *P* < 0.05 were PRR11, age T stage, N stage and M stage in the Cox univariate regression model. Multivariate analysis further demonstrated that PRR11 and N stage were independent prognostic factors in OS of BLCA patients (Fig. 3b).

### Correlations of PRR11 expression with tumor mutation burden, tumor microsatellite instability and mismatch repair genes

**Figure 4:**
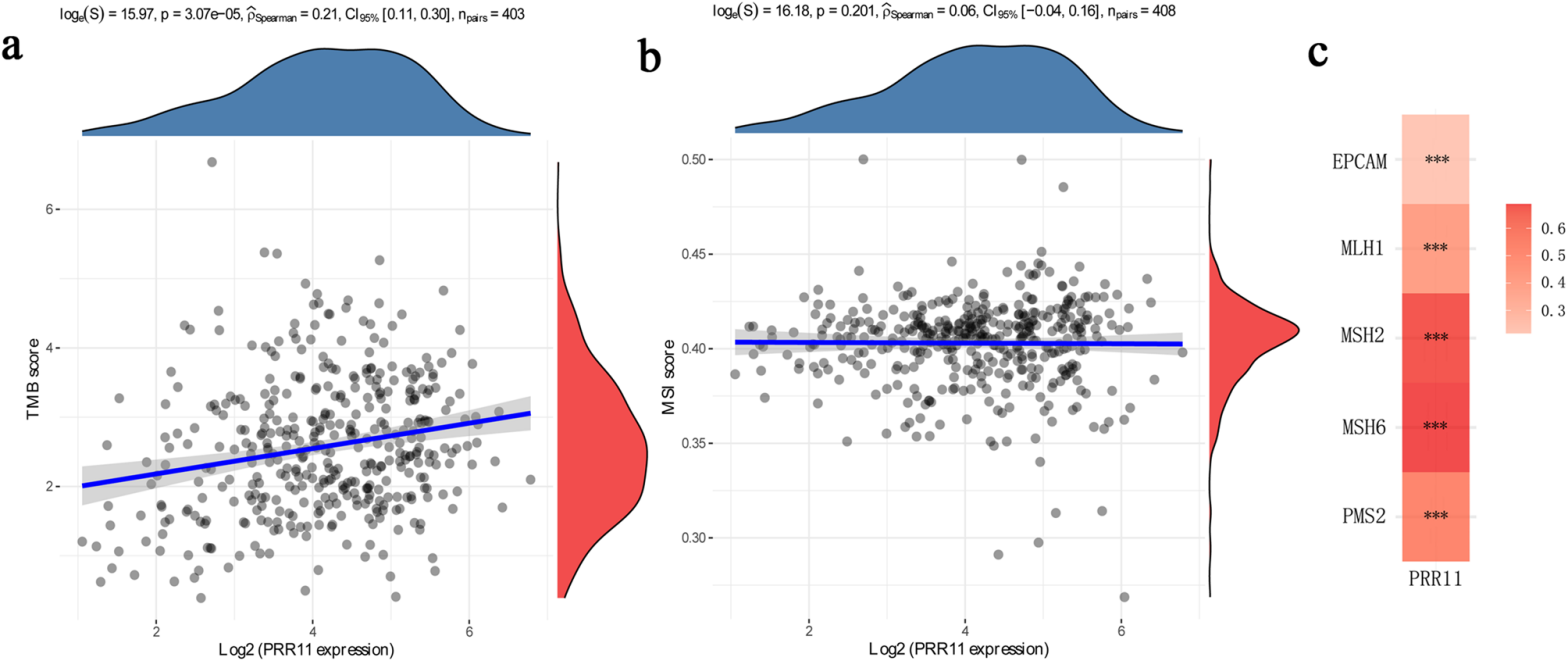
Correlation analysis of PRR11 expression and TMB (**a**)/MSI (**b**). The horizontal axis in the figure represents the expression distribution of the gene, and the ordinate is the expression distribution of the TMB/MSI score. The density curve on the right represents the distribution trend of the TMB/MSI score; the upper density curve represents the distribution trend of the gene; the value on the top side represents the correlation p value, correlation coefficient and correlation calculation method. (**c**) Heatmap illustrating the association between PRR11 expression and MMR genes. The gene correlation heatmap is realized by the R (v4.1.0) software package “ggstatsplot”. ***P < 0.001.

Subsequently, considering the sensitivity of clinical immunotherapy, we investigated the correlation between the expression level of PRR11 and TMB, MSI, and MMR-related genes (MLH1, MSH2, MSH6, PMS2, and EPCAM). The results indicated that the expression of PRR11 in BLCA was positively correlated with TMB (Fig. 4a) but not significantly correlated with MSI (Fig. 4b). MMR gene expression is obviously and significantly positively correlated with PRR11 levels (Fig. 4c).

### PRR11 coexpression network in BLCA

**Figure 5:**
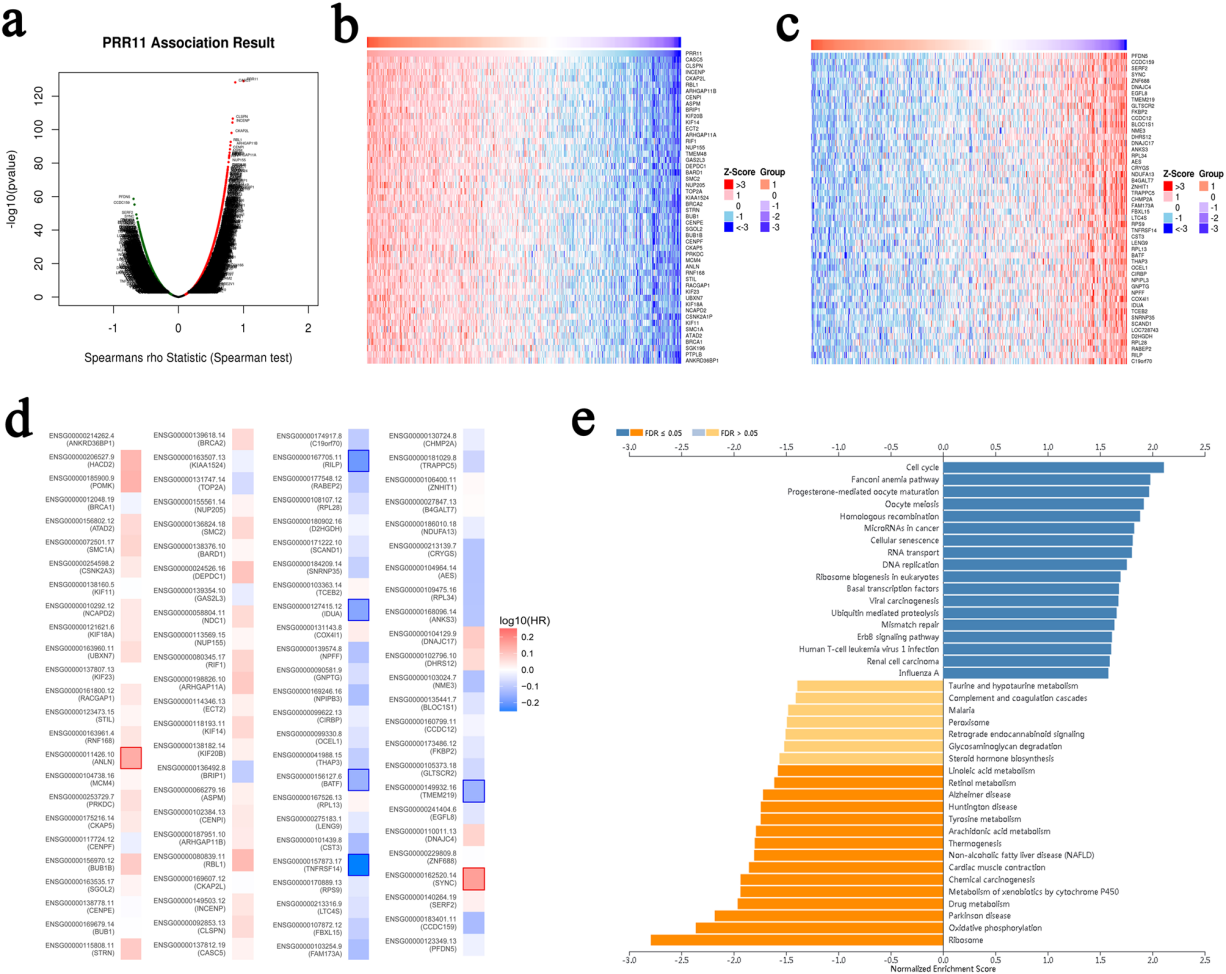
PRR11 coexpression network in BLCA from LinkedOmics database. (**a**) All genes associated with PRR11 in BLCA cohort, red dots positively correlated with PRR11, and green dots negatively correlated. Heat map of the top 50 genes positively (**b**) and negatively (**c**) correlated with PRR11 in BLCA. Red represents positively linked genes and blue represents negatively linked genes. The gene expression heatmap is realized by LinkedOmics database (http://www.linkedomics.org/login.php). (**d**) Survival map of the top 50 genes positively and negatively associated with PRR11 in BLCA. The survival heatmap is realized by GEPIA Database (http://gepia.cancer-pku.cn/index.html). (**e**) PRR11 of KEGG pathway enrichment analysis in BLCA cohort.

To further clarify the biological role of PRR11 in BLCA, we used the LinkedOmics database to explore the coexpression characteristics of PRR11 based on the BLCA samples from TCGA. The scatter plot illustrates that PRR11 is correlated with a total of 20,047 genes, of which 10,074 genes are positively correlated with it, and the remaining 9,973 genes are negatively correlated with it (Fig. 5a). We selected the top 50 genes that were positively and negatively associated with PRR11, and demonstrated their expression pattern as a heatmap (Fig. 5b,c). Further analysis of these 100 genes revealed that 37 of 50 positive correlation genes had unfavorable HR, and one was statistically significant (ANLN). In contrast, 34/50 genes negatively related to PRR11 had the possibility of being potential protective markers in BLCA patients, and 5 of them were statistically significant (IDUA, BATF, TNFRSF14, RILP, TMEM219). It was worth mentioning that SYNC, as a gene negatively correlated with PRR11, had unfavorable HR (Fig. 5d). The results of KEGG pathway enrichment analysis demonstrated that PRR11 co-expressed genes were mainly involved in the cell cycle, homologous recombination, microRNAs in cancer, RNA transport, DNA replication, and ErbB signaling pathway (Fig. 5e). GO term annotation (biological process, cellular component and molecular function) demonstrated that co-expressed genes of PRR11 are primarily associated with chromosome segregation, DNA replication, cell cycle, double-strand break repair, spindle organization and a series of biological processes surrounding nucleic acid, and also related to enzyme activities, such as Helicase and ATPase. (Supplementary Fig. 2). These findings imply that the PRR11 co-expression network may play an important role in the prognosis, initiation and progression of BLCA, but further follow-up studies are needed.

### Association between PRR11 with immune infiltration

**Figure 6:**
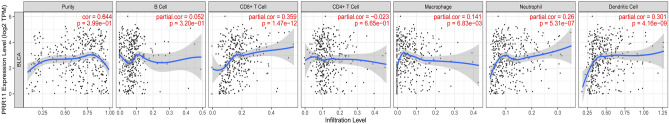
Correlation of PRR11 expression with immune infiltration level in BLCA.

**Figure 7:**
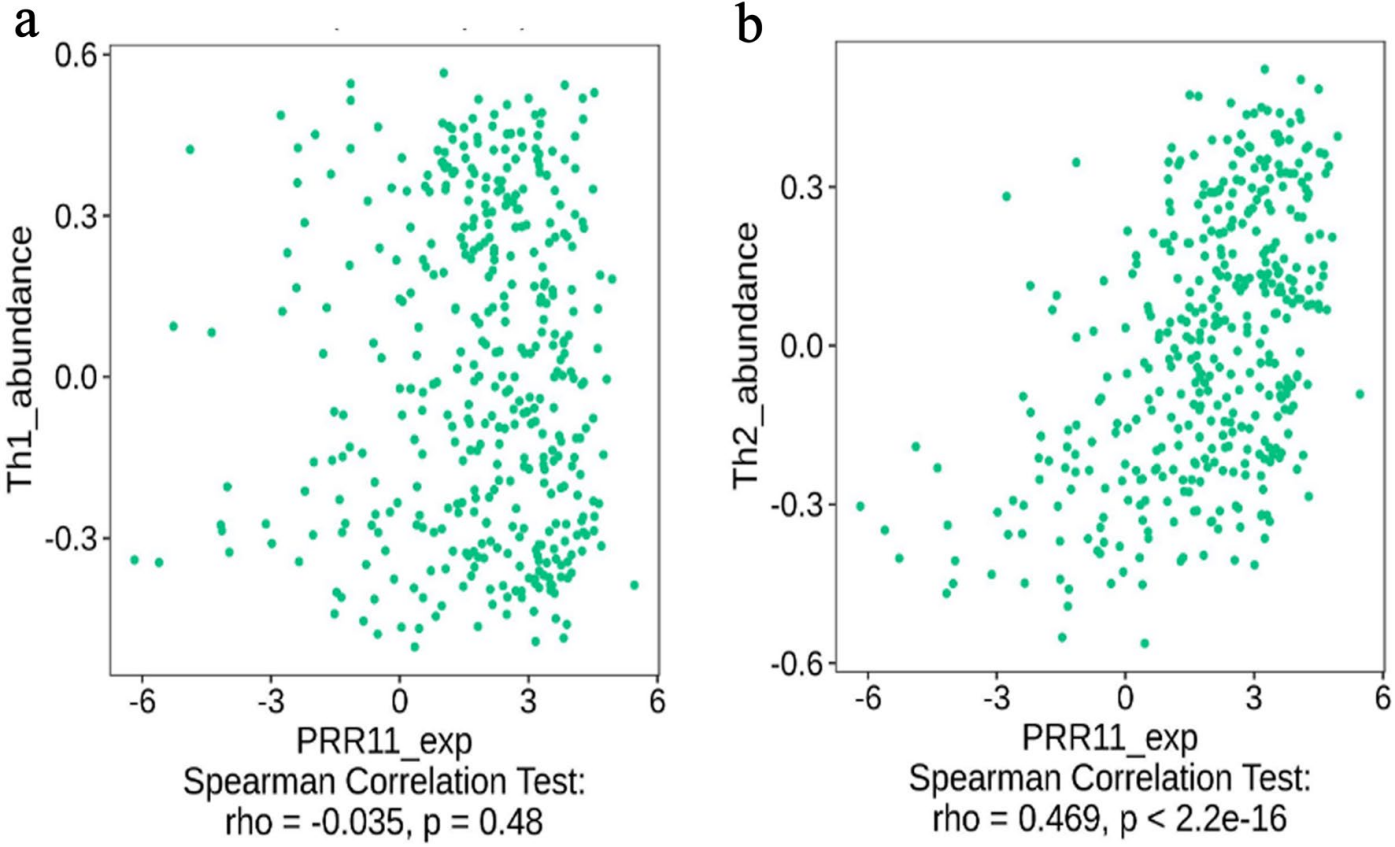
Correlation of PRR11 expression with infiltration level of Th1 and Th2 in BLCA from TISIDB database.

We used the TIMER database to investigate the relationship between PRR11 and the level of immune infiltration in tumors, given the significance of this parameter. The results revealed that the expression level of PRR11 and the infiltration level of CD8^+^ T cell, macrophage, neutrophil , and dendritic cells in BLCA were significantly positively correlated (Fig. 6). By analyzing the TISIDB database, we also found that the infiltration level of Th2 cell was significantly positively correlated with the PRR11 expression, whereas Th1 did not have any significant correlations with the expression of PRR11 (Fig. 7a,b).

**Figure 8:**
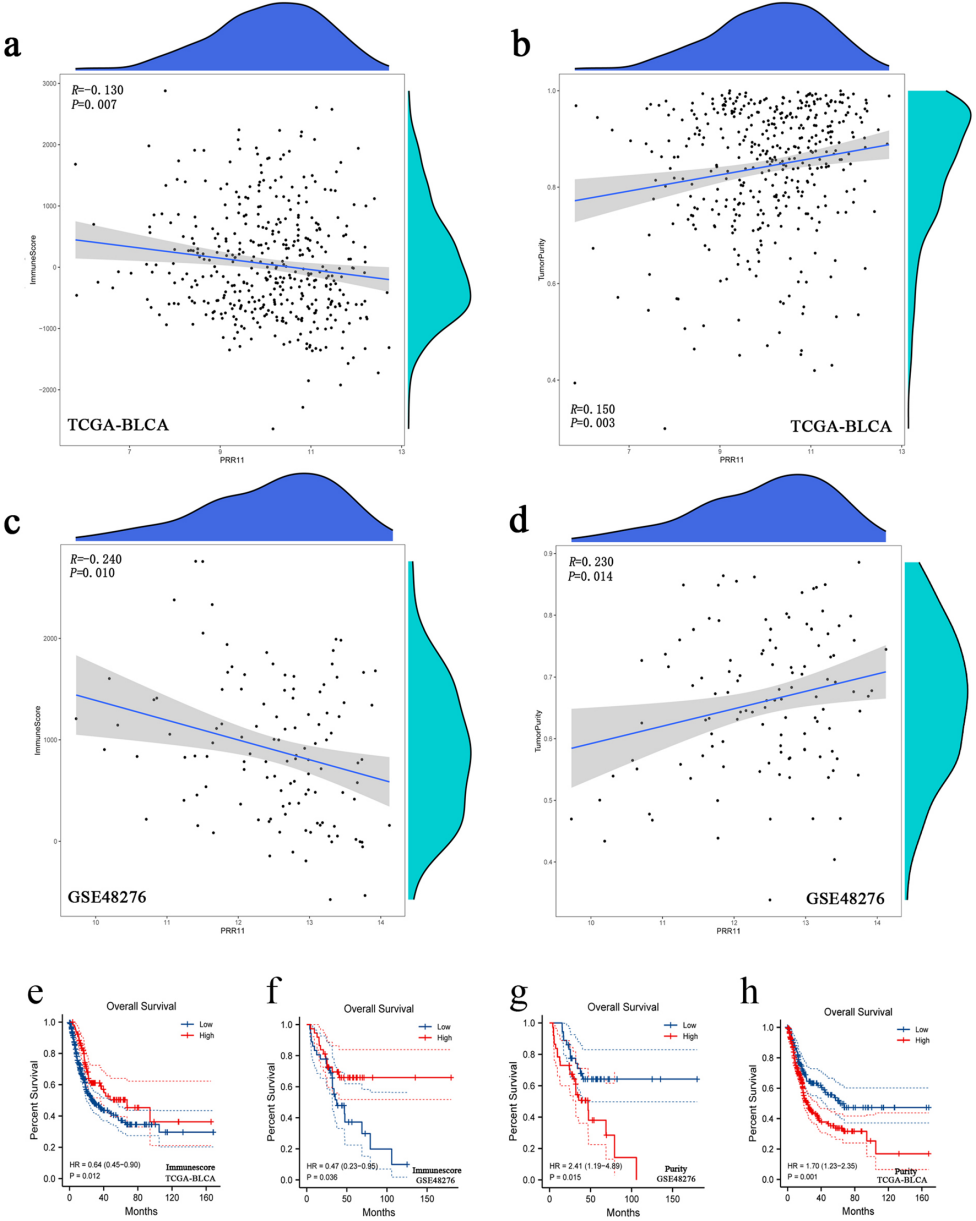
Associations between the PRR11 expression and immune infiltration level in BLCA from the ESTIMATE algorithm. (**a**–**d**) The expression of PRR11 has a significant negative correlation with the immunescore and a positive correlation with tumorpurity score of BLCA samples based on the ESTIMATE algorithm in the TCGA-BLCA and GSE48276 dataset. The horizontal axis in the figure represents gene expression, and the vertical axis is purity/immunescore. The density curve on the right represents the distribution trend of purity/immunescore; the upper density curve represents the distribution trend of genes. (**e**–**h**) Kaplan–Meier survival curves were used to analyze the correlation between purity/immunescore and OS of BLCA patients. Patients with higher immunescore and lower tumorpurity score have higher OS time in the TCGA-BLCA and GSE48276 dataset.

Then, we used the ESTIMATE algorithm to determine whether the expression of PRR11 was associated with the infiltration level of total immunity in BLCA. The calculation results demonstrated that in the two datasets of TCGA-BLCA and GSE48276, PRR11 and Immunescore had a significant negative correlation, and a significant positive correlation with tumor purity (Fig. 8a–d). The prognostic analysis also revealed that patients with high immune scores had better overall survival time than patients with low scores, whereas patients with high tumor purity scores had a worse prognosis (Fig. 8e–h), which was consistent with the prognostic results of single-factor calculations that included only PRR11 expression.

**Figure 9:**
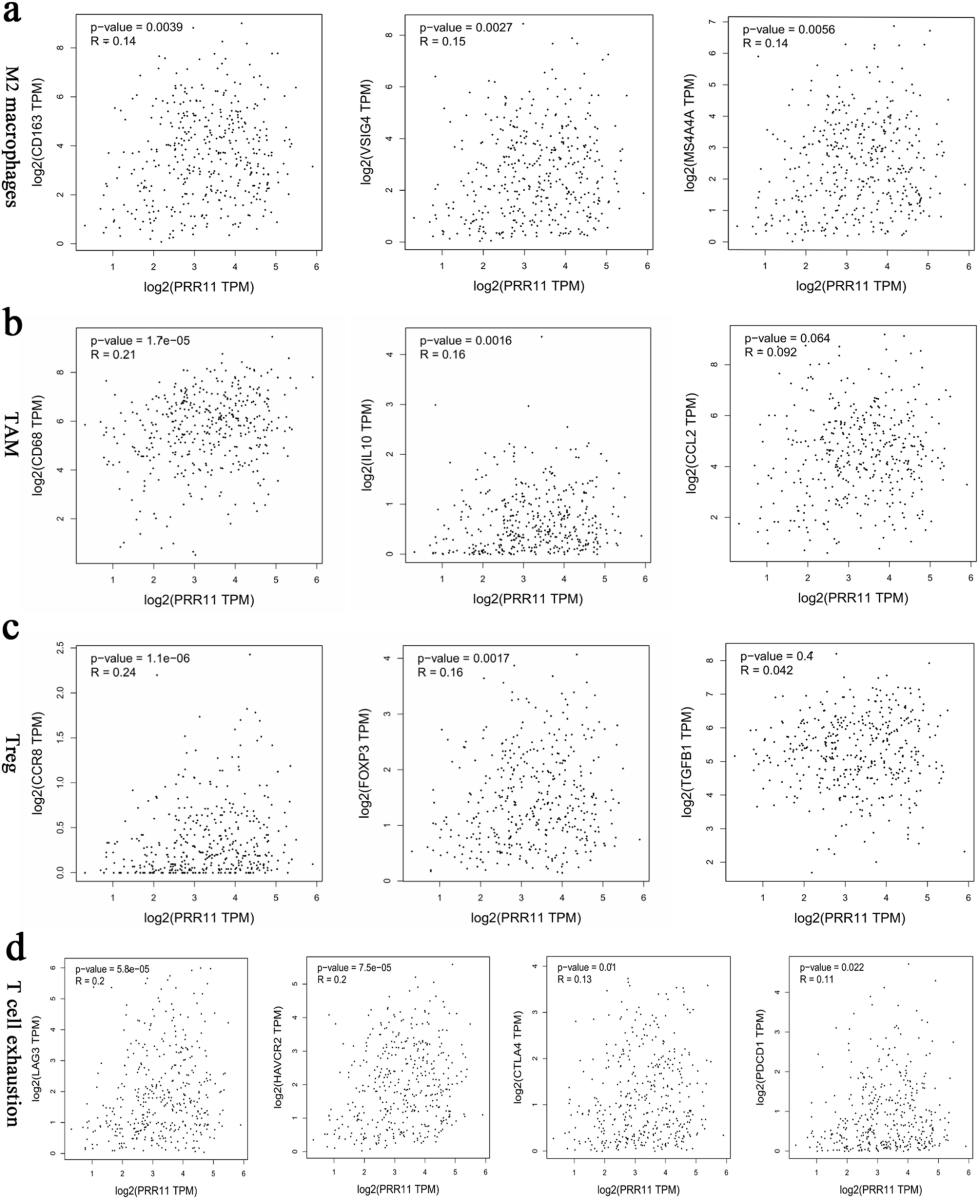
(**a**) Scatterplots of correlations between PRR11 expression and gene markers of M2 macrophages. (**b**) Scatterplots of correlations between PRR11 expression and gene markers of TAM. (**c**) Scatterplots of correlations between PRR11 expression and gene markers of T cell exhaustion.

To gain a deeper understanding of the potential association between PRR11 and immune infiltration, we also analyzed the correlation between the expression of PRR11 and immune marker sets of various immune cells in BLCA using the GEPIA database. Interestingly, the data demonstrated a significant correlation between PRR11 and a large number of immune cell-related molecular markers, such as marker sets of monocyte, neutrophil, natural killer cell , and CD8 + T cell (Table 3). In particular, it should be pointed out that these main roles in regulating tumor immune microenvironment, such as marker sets related to M2 macrophage, tumor-associated macrophages (TAM), Treg and T cell exhaustion, displayed a strong and significant positive correlation with PRR11 (Table 3, Fig. 9a–d). Therefore, we hypothesize that PRR11 indirectly affects the negative regulation of the tumor immune microenvironment and influences the phenotype of BLCA by regulating immune cell infiltration.

**Table 3:** Correlation analysis between PRR11 and relate genes of immune cells. *P < 0.05; **P < 0.01; ***P < 0.001.

Description	Gene makers	BLCA	
		R	P
M1 Macrophages	NOS2	0.14	**
	PTGS2	0.09	0.071
	IRF5	− 0.037	0.45
M2 Macrophages	CD163	0.14	**
	VSIG4	0.15	**
	MS4A4A	0.14	**
TAM	CCL2	0.092	0.064
	CD68	0.21	***
	IL10	0.16	**
Monocyte	CD86	0.2	***
	CD115	0.13	**
Neutrophils	CD11B	0.17	***
	CCR7	0.19	***
Dendritic cell	HLA-DPB1	0.059	0.23
	HLA-DRA	0.11	0.021
	CD11C	0.17	***
Natural killer cell	KIR2DL1	0.067	0.18
	KIR2DL3	0.16	**
	KIR3DL2	0.19	***
CD8^+^ T cell	CD8A	0.15	**
	CD8B	0.12	*
Th1	T-bet	0.012	0.82
	STAT4	0.083	0.097
	STAT1	0.24	***
	TNF-A	0.047	0.35
	IFN-γ	0.047	0.34
Th2	GATA3	− 0.2	***
	STAT6	− 0.018	0.71
	IL13	− 0.005	0.92
Tfh	BCL6	0.0068	0.89
	IL21	0.14	**
Th17	STAT3	0.43	***
	IL17A	− 0.082	0.1
Treg	FOXP3	0.16	**
	CCR8	0.24	***
	TGF-β	0.0042	0.4
T cell exhaustion	PD-1 (PDCD1)	0.11	*
	CTLA4	0.13	*
	LAG3	0.2	***
	TIM3 (HAVCR2)	0.2	***
B cell	CD19	0.011	0.83
	CD79A	− 0.059	0.24

## Discussion

Bladder cancer is one of the three major malignant tumors of the genitourinary system. However, the current diagnosis and treatment methods for this disease are limited, so exploring novel tumor markers and therapeutic targets is a significant direction in this field. PRR11 is a relatively new potential candidate oncogene, which may play an important role in the initiation and progression of cancer. Several studies have reported that PRR11 overexpression promotes the proliferation, migration, and invasion of ovarian cancer cells by activating the PI3K/AKT/β-Catenin pathway^24^, and it also can promote the progression of breast cancer by activating EMT^39^. In addition, PRR11 silencing not only causes cell cycle arrest, inhibits colony formation, reduces cell proliferation, and inhibits the tumorigenic potential of lung cancer cells^40^, but also inhibits the growth and EMT of liver cancer cells through β-catenin signaling^20^. PRR11 is also considered a potential new target for the diagnosis and treatment of lung cancer^41^.

Transcriptome analysis of clinical samples from databases such as TCGA and Oncomine demonstrated that the level of PRR11 mRNA in BLCA was significantly higher than that in non-cancerous tissues, regardless of whether it was observed from the overall sample or the matched sample of a single patient. By analyzing the HPA database, we confirmed the high expression status of PRR11 in BLCA patients at the protein level. In addition, we found that the expression level of PRR11 significantly affected the pathological staging of tumors. Moreover, the multivariate analysis revealed that PRR11 expression was an independent prognostic factor of BLCA patients. Therefore, our findings suggest that PRR11 is up-regulated in the vast majority of BLCA samples and is involved in the pathological process of BLCA tissues. As a potential prognostic marker, PRR11 deserves more detailed clinical verification.

The analysis using the LinkedOmics database revealed that most genes co-expressed with PRR11 in BLCA exhibited prognostic trends, and six genes were statistically significant in BLCA patients. Moreover, these co-expressed genes were highly concentrated in the cell cycle, DNA repair and replication, cancer-related microRNA, ErbB signaling pathway, and other tumor-related biological pathways, consistent with the known function of PRR11. Future research on PRR11 in BLCA may reveal the relevant molecular mechanism.

TMB is a promising biomarker for cancer prognosis that can direct immunotherapy in the era of precision medicine^44^. Previous studies have demonstrated that TMB can be used as a biomarker to improve immunotherapy^45^. In addition, MSI is an important biomarker for immune checkpoint inhibitors (ICI)^46^. According to our research, the PRR11 expression in BLCA positively correlates with TMB but does not significantly correlate with MSI. This may indicate that the expression level of PRR11 affects the TMB of BLCA, thereby influencing the patient's response to immune checkpoint suppression therapy. Additionally, we observed a positive correlation between PRR11 expression and MMR gene expression. Therefore, patients with high PRR11 expression and TMB levels may have better prognoses following ICI treatment in BLCA.

It is well known that the immune environment constructed by multiple immune cells as part of TME has been considered critical to the progression of tumors and the overall effectiveness of cancer treatments, including chemotherapy and radiotherapy, especially immunotherapy^13,14,42,43^. According to the calculation results of the ESTIMATE algorithm, PRR11 expression was significantly correlated with immune score and tumor purity scores in BLCA samples, and patients grouped by immune score or tumor purity scores also had a constant prognostic relationship with patients grouped with PRR11 expression. These findings clearly show that increasing the level of immune cell infiltration and decreasing the expression of PRR11 may improve the tumor immune microenvironment and prognosis of BLCA patients.

The TIMER database analysis visually revealed that the expression of PRR11 is correlated with the immune cell infiltration in BLCA. In addition, the results from the TISIDB database and GEPIA database demonstrated that the PRR11 in BLCA was positively correlated with the Th2, CD8 + T cells, M2 macrophages, TAM, Treg, NK, neutrophils, monocytes, and the markers of T cell exhaustion, such as PDCD1, TIM3, CTLA4, and LAG3.

Macrophages in the tumor immune microenvironment tend to become TAM, which is differentiated from monocytes migrating to tumor stroma to drive tumor progression, invasion, and metastasis^47,48^. The relationship between PRR11 and monocytes is supported by TAM data in our findings. In addition, reports indicate that Th1 cytokines activate M1 macrophages to exert anti-tumor effects, whereas Th2 cytokines can activate M2 macrophages to exert tumor-promoting effect^49^. Although CD4 + T cells and PRR11 did not show a significant correlation in our analysis results, we speculate that PRR11 in BLCA may not affect the total CD4 + T cell infiltration level, but it can regulate the subtypes of infiltrating immune cells. This means that PRR11 increases the proportion of Th2 cells to produce an immunosuppressive TME. However, considering that the correlation between PRR11 and Th2-related molecular markers was insignificant, we believe there may be other indirect control methods. The literature illustrates that tumor-infiltrating dendritic cells also tend to promote immune suppression and tolerance rather than driving anti-tumor immunity^50^, and some dendritic cells are derived from monocytes (moDCs)^51^. Neutrophils in tumor tissues typically transform into N2 subtypes with pro-tumor effects^50^.

Tregs promote tumor growth and expansion by supporting the host's immune response, accelerating angiogenesis, and tissue remodeling^52^. Especially functional FOXP3^+^ Treg cells can inhibit cytotoxic CD8^+^ T cells from attacking tumor cells and promote tumor development^53^.

Finally, it should be pointed out that CD8^+^ T cells are an independent prognostic factor in certain cancers, such as pancreatic cancer, and they can play a tumor suppressor role in the tumor immune microenvironment^54–57^. Although there was a significantly positive correlation between PRR11 and CD8^+^ T cells in BLCA, it could not be ignored that it also demonstrated a broad positive correlation with the four molecular markers of T cell exhaustion (PDCD1, CTLA4, LAG3, TIM3). Based on this, we believe that PRR11 may increase the level of CD8^+^ T cell infiltration in BLCA tumor tissues to a certain extent, but these tumor-infiltrating CD8 + T cells cannot exert their ability of tumor suppressor due to T cell exhaustion and the suppressive immune microenvironment. These findings also highlight the complex mechanism of PRR11 in regulating the tumor immune microenvironment.

Therefore, we suggest that PRR11 in BLCA tends to induce a tumor-suppressive immune microenvironment, and the up-regulation of its expression may directly or indirectly alter the type and level of immune cell infiltration. In this way, the tumor immune microenvironment of BLCA patients is altered to a tumor-promoting type, which has a negative impact on patient prognosis.

In conclusion, PRR11 has the potential as a molecular marker for the poor prognosis of BLCA. Increased expression of PRR11 is associated with deterioration of clinical characteristics, such as tumor pathological grade and prognosis (OS and DFS). In addition, the expression of PRR11 in BLCA is significantly related to tumor immune infiltration, which will inevitably have a profound effect on the prognosis of BLCA. Moreover, given the relationship between PRR11 and the TMB and MMR genes, it is reasonable to assume that PRR11 has considerable value in ICI. Therefore, in the future, we can try to evaluate the prognosis and risk stratification of patients by detecting the expression level of PRR11 in surgical specimens from BLCA patients, as well as develop immunotherapy drugs targeting PRR11 based on the immune microenvironment.

## Supplementary Information

Supplementary Information.

## Data Availability

All relevant data are within the paper.

## Abbreviations

PRR11: Proline-rich protein
BLCA: Bladder urothelial carcinoma
TCGA: The Cancer Genome Atlas
GEO: Gene Expression Omnibus
TMB: Tumor mutational burden
MSI: Microsatellite instability
MMR: Mismatch repair
MLH1: MutL homologous gene
MSH: MutS homologous gene
PMS2: Postmeiotic segregation increased 2
EPCAM: Epithelial cell adhesionmolecule
TURB: Transurethral resection
TME: Tumor microenvironment
EMT: Epithelial-mesenchymal transition
FPKM: Fragments per kilobase per million
GTEx: Genotype-Tissue Expression
ROC: Receiver operating characteristic
AUC: Area under curve
HPA: Human Protein Atlas
GEPIA: Gene expression profiling interactive analysis
OS: Total survival
TIMER: Tumor IMmune Estimation Resource
KEGG: Kyoto Encyclopedia of Genes and Genomes
GSEA: Gene set enrichment analysis
RFS: Relapse-free survival
TAM: Tumor-associated macrophages
moDCs: Dendritic cells are derived from monocytes
