# Supplementary material for: PRR11 is a prognostic biomarker and correlates with immune infiltrates in bladder urothelial carcinoma
Source: Sci Rep. 2023 Feb 4;13:2051. doi: 10.1038/s41598-023-29316-2 (PMC9899238; doi:10.1038/s41598-023-29316-2)
Supplement: Supplementary file 1 — Supplementary Information. [file 41598_2023_29316_MOESM1_ESM.docx]

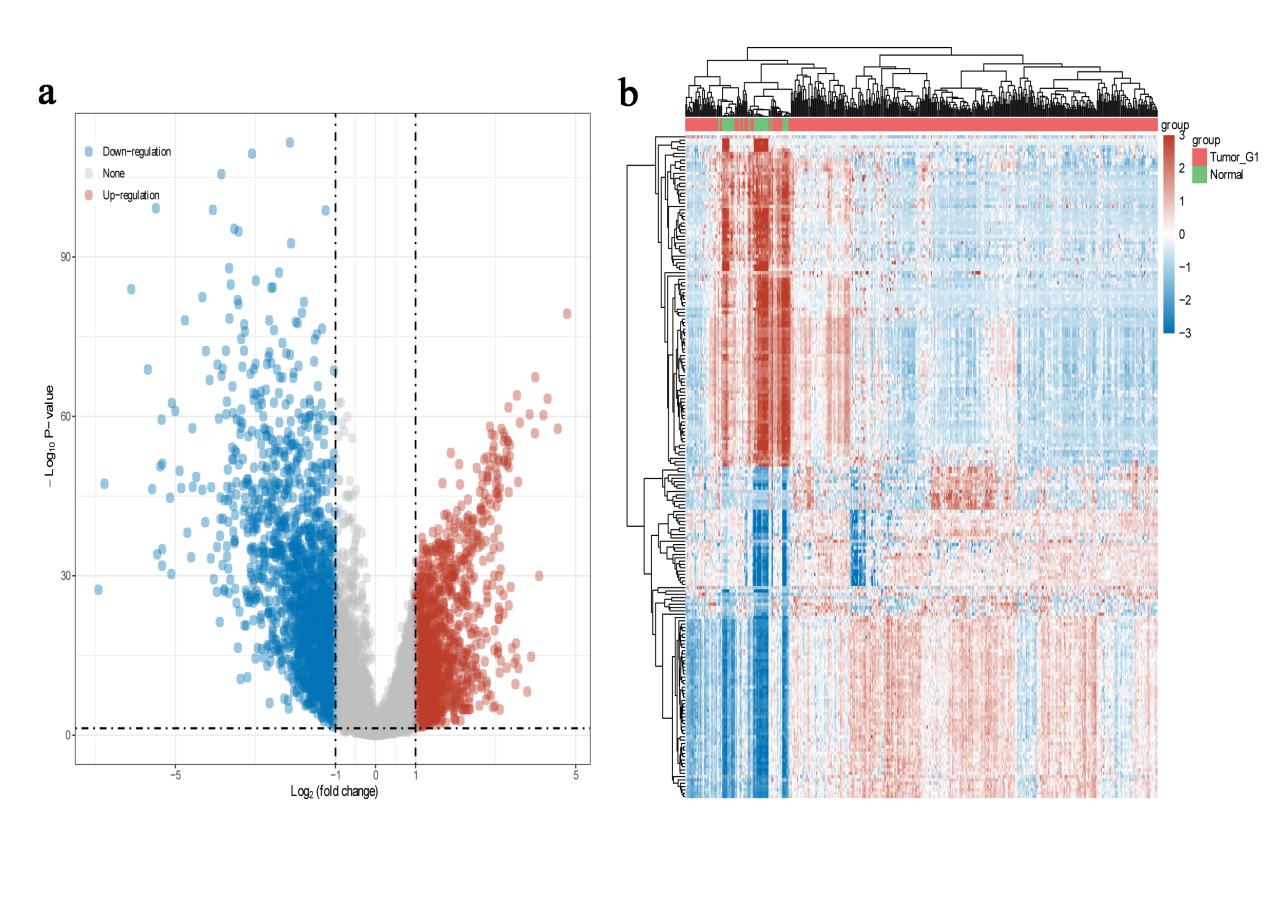


Supplementary Fig. 1 Genome-wide analysis of different gene in BLCA. (a) Volcano plots were constructed using fold-change values and adjusted P. The red point in the plot represents the over-expressed mRNAs and the blue point indicates the down-expressed mRNAs with statistical significance. (b) Hierarchical clustering of mRNAs, which were differentially expressed between tumor and normal tissue. The 50 most differentially up-regulated and 50 down-regulated genes are shown here.


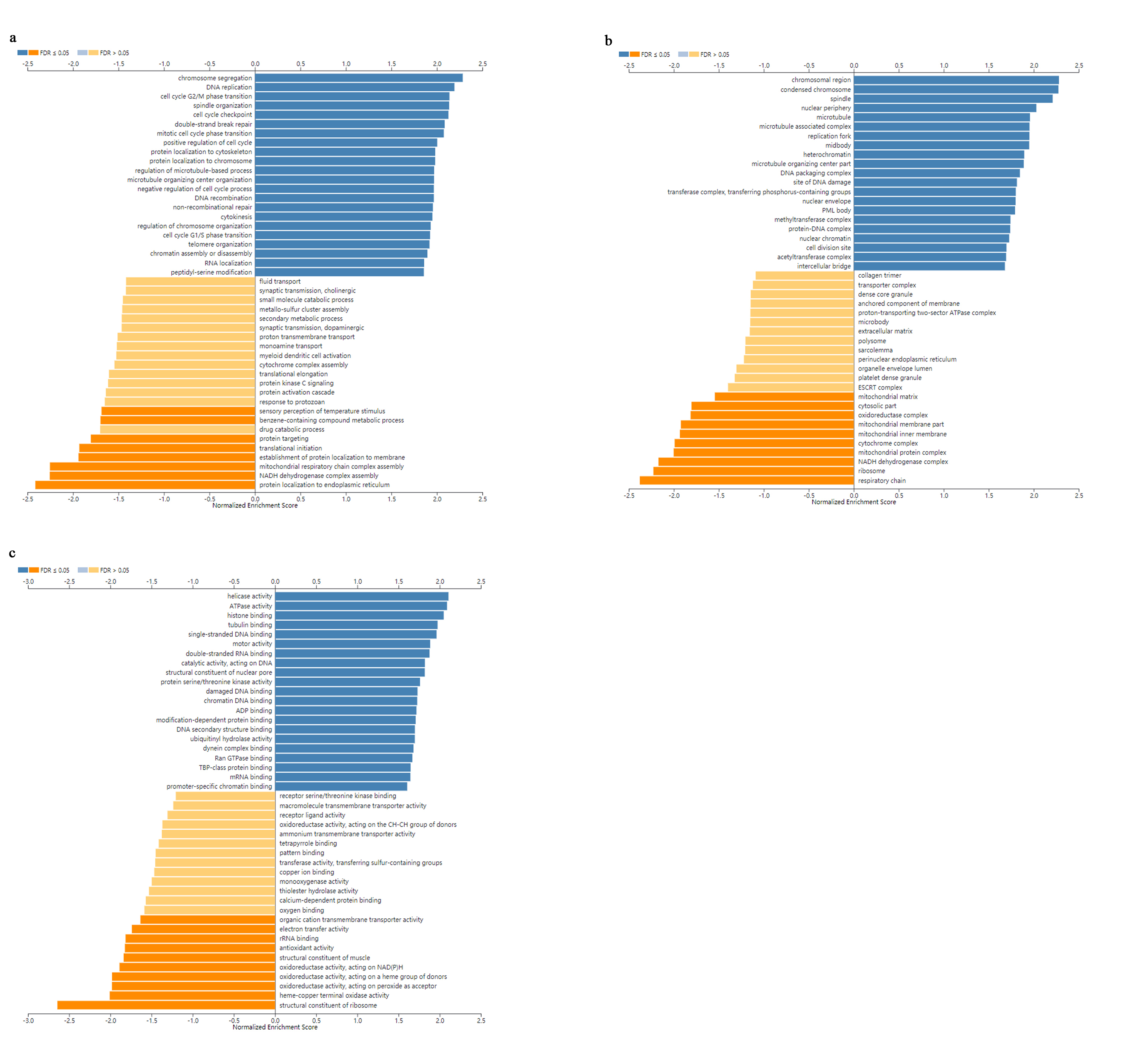


Supplementary Fig. 2 GO annotations of PRR11 in BLCA cohort. (a) Biological process; (b) Cellular component; (c) Molecular function.
